# Oxidative Stress, DNA Damage and DNA Repair in Female Patients with Diabetes Mellitus Type 2

**DOI:** 10.1371/journal.pone.0162082

**Published:** 2016-09-06

**Authors:** Annemarie Grindel, Bianca Guggenberger, Lukas Eichberger, Christina Pöppelmeyer, Michaela Gschaider, Anela Tosevska, George Mare, David Briskey, Helmut Brath, Karl-Heinz Wagner

**Affiliations:** 1 Department of Nutritional Sciences, Emerging Field Oxidative Stress and DNA Stability, University of Vienna, Vienna, Austria; 2 Research Platform Active Ageing, University of Vienna, Vienna, Austria; 3 School of Human Movement and Nutrition Sciences, University of Queensland, St Lucia, QLD, Australia; 4 Diabetes Outpatient Clinic, Health Centre South, Vienna, Austria; Virgen Macarena University Hospital, School of Medicine, University of Seville, SPAIN

## Abstract

**Background:**

Diabetes mellitus type 2 (T2DM) is associated with oxidative stress which in turn can lead to DNA damage. The aim of the present study was to analyze oxidative stress, DNA damage and DNA repair in regard to hyperglycemic state and diabetes duration.

**Methods:**

Female T2DM patients (n = 146) were enrolled in the MIKRODIAB study and allocated in two groups regarding their glycated hemoglobin (HbA1c) level (HbA1c≤7.5%, n = 74; HbA1c>7.5%, n = 72). In addition, tertiles according to diabetes duration (DD) were created (DDI = 6.94±3.1 y, n = 49; DDII = 13.35±1.1 y, n = 48; DDIII = 22.90±7.3 y, n = 49). Oxidative stress parameters, including ferric reducing ability potential, malondialdehyde, oxidized and reduced glutathione, reduced thiols, oxidized LDL and F2-Isoprostane as well as the activity of antioxidant enzymes superoxide dismutase, catalase and glutathione peroxidase were measured. Damage to DNA was analyzed in peripheral blood mononuclear cells and whole blood with single cell gel electrophoresis. DNA base excision repair capacity was tested with the modified comet repair assay. Additionally, mRNA expressions of nine genes related to base excision repair were analyzed in a subset of 46 matched individuals.

**Results:**

No significant differences in oxidative stress parameters, antioxidant enzyme activities, damage to DNA and base excision repair capacity, neither between a HbA1c cut off />7.5%, nor between diabetes duration was found. A significant up-regulation in mRNA expression was found for *APEX1*, *LIG3* and *XRCC1* in patients with >7.5% HbA1c. Additionally, we observed higher total cholesterol, LDL-cholesterol, LDL/HDL-cholesterol, triglycerides, Framingham risk score, systolic blood pressure, BMI and lower HDL-cholesterol in the hyperglycemic group.

**Conclusion:**

BMI, blood pressure and blood lipid status were worse in hyperglycemic individuals. However, no major disparities regarding oxidative stress, damage to DNA and DNA repair were present which might be due to good medical treatment with regular health checks in T2DM patients in Austria.

## Introduction

Diabetes mellitus type 2 (T2DM) with its resulting complications is one of the biggest preventable health problems of the 21^st^ century and has developed to a major challenge for the health system as one of the fastest increasing diseases worldwide [1]. T2DM develops mostly undiagnosed in overweight individuals as a result of pancreatic ß-cell dysfunction and impaired glucose tolerance [2, 3]. Untreated, the underlying hyperglycemia leads to a ß-cell failure and an indispensable need for exogenous insulin supply [4, 5]. Hyperglycemia is usually measured by the percentage of glycated hemoglobin (HbA1c) from the total amount of blood hemoglobin, which evolves through a long-term exposure to elevated glucose in the blood stream [6]. Chronic hyperglycemia promotes oxidative stress [7, 8] which represent a major pathophysiological link between progression of T2DM and the onset of severe diabetic complications such as diabetic foot ulcers, myocardial infarction or cerebrovascular accidents [9]. Especially cardiovascular events lead to premature mortality in diabetes patients [10]. Furthermore, oxidative stress can trigger damage to DNA which has been linked to enhanced cancer risk [11]. Therefore patients with diabetes mellitus show an increased cancer incidence, with a strong linear association between HbA1c levels and gastric-, pancreatic-, colorectal-, breast- and liver cancer incidence [12, 13].

Damage to DNA does not necessarily lead to severe complications with a phenotypic outcome. In most cases, DNA repair mechanisms, including base excision repair (BER), nucleotide excision repair, direct reversal repair, mismatch repair, homologous recombination, non-homologous end joining and translesion synthesis [14], cope with this damage and maintain the cells’ homeostasis. BER is most efficient in repairing endogenous DNA damage employing DNA glycosylases, such as mutY DNA glycosylase (*MUTYH*), nth-like DNA glycosylase 1 (*NTHL1*), 8-oxoguanine DNA glycosylase (*OGG1*) or the nei-like DNA glycosylase 1 (*NEIL1*) to excise aberrant bases (either purins or pyrimidines). The resulting apurinic-/ apyrimidinic (AP) sites are recognized by AP endonucleases (*APEX1*) initiating repair by cleaving the sugar-phosphate backbone. Finally, DNA polymerase beta (*POLB*) fills in the gap and builds a complex with X-ray repair cross-complementing protein 1 (*XRCC1*) and DNA ligase 3 (*LIG3*) to insert the correct complementary base in the AP site [15]. In T2DM the DNA repair system is reported to be down-regulated [16, 17], while DNA damage [18, 19] and oxidative stress parameters [20–24] accumulate.

Most of the aforementioned studies compared T2DM to healthy controls. However, within its progression T2DM is a considerably diverse disease requesting different medical treatment approaches leading to a broad range of hyperglycemia. Our intention for this cross-sectional study was therefore not to compare T2DM patients to healthy controls, but rather to compare well-controlled patients with HbA1c≤7.5% to individuals with HbA1c>7.5% and patients with a short diabetes duration (DD) to persons with longer DD, in regard to oxidative stress, antioxidant enzyme activities, DNA damage and DNA repair.

## Materials and Methods

### Study population

The cross-sectional human MIKRODIAB study was performed in 2014 at the local Diabetes Outpatient Clinic (Health Centre South, Austria, Vienna 1100) in cooperation with the Department of Nutritional Sciences of the University of Vienna. A total of 154 female patients with T2DM were recruited during their regular health assessment in respect to the inclusion criteria: female gender, age above 30 years, oral antidiabetics and/or insulin therapy as medication, constant nutritional behavior, constant physical activity, constant weight for the last 4 weeks, non-smoking for at least 1 year. Additionally, exclusion criteria of the study were: pregnancy or lactation; change of medication in regard to metabolic parameters within the last 4 weeks; cardiovascular damage with NYHA>III; liver disease with three-times higher transaminase values; chronic kidney disease with serum creatinine>2 mg/dl; dialysis; HIV positive; history of chronic alcohol abuse in the last two years; history of cancer, stroke or organ transplantation. Eight patients did not fulfill these criteria and were excluded from the study population. The resulting 146 female T2DM volunteers were assigned to two groups in regard to their HbA1c level (HbA1c≤7.5%, n = 74; HbA1c>7.5%, n = 72). In addition, three groups according to diabetes duration (DD) were created (DD I = 6.94±3.1 y, n = 49, DD II = 13.35±1.1 y, n = 48, DD III = 22.90±7.3 y, n = 49).

The study was approved by the Ethics Committee of the Medical University of Vienna (EK Nr: 1987/2013) and was performed in accordance to the Declaration of Helsinki. All subjects gave their written consent. The study has been registered at ClinicalTrials.org (NCT02231736).

### Sample collection and blood sample preparation

Fasting blood was sampled by venipuncture at the Diabetes Outpatient Clinic during the next regular health check of the patients. In total 45 ml EDTA-blood (Vacuette, K2EDTA, Greiner Bio-one GmbH) and 5 ml whole blood for serum isolation (Vacuette, Z Serum Sep, Greiner Bio-one GmbH) were collected. For anthropometric assessment, body height (stadiometer: model 214, Seca), weight (scale: selecta 791, Seca), blood pressure (Boso medicus control, Bosch + Sohn GmbH), waist circumference and hip circumference were measured at the study day. Body mass index (BMI) was calculated as kg/m². Additionally, nutritional behavior, physical activity, medical history, socio-economic status and life quality were assessed by questionnaires.

Blood samples were processed immediately. For detection of DNA damage, whole blood aliquots (a 100 μl) were gradually frozen to -80°C (CoolCell, Biozym). EDTA-plasma, for analyses of malondialdehyde (MDA) and oxidized low-density lipoprotein (oxLDL) as well as serum for measurement of reduced thiols and antioxidant capacity were aliquoted and stored at -80°C. Serum samples for analysis of oxidized (GSSG) and reduced (GSH) glutathione were treated with 10% tetraacetic acid before storage as described by Boon et al. [25]. The isolation of peripheral blood mononuclear cells (PBMC) was performed with density gradient centrifugation using Leucosep tubes (Greiner Bio-one) as described earlier [26]. For storage, viable PBMC were resuspended in freezing media, containing fetal bovine serum with 10% dimethyl sulfoxid, and gradually frozen (CoolCell, Biozym). For detection of DNA BER capacity, 5x10^6^ PBMC were taken from each participant for extract preparation. The cell pellet was snap frozen in liquid nitrogen and stored until analyses. PBMC for gene expression analyses were stored in RNA*later* (Sigma-Aldrich Co.) until RNA isolation. All samples were stored at -80°C until analyses.

### Analyses of biochemical parameters

The biochemical parameters HbA1c, fasting plasma glucose, fasting plasma insulin, C-peptide, total cholesterol, triglycerides, high-density lipoprotein (HDL)-cholesterol and low-density lipoprotein (LDL)-cholesterol were measured immediately after blood sampling at the laboratory of the Diabetes Outpatient Clinic as described previously [26, 27]. Homeostasis model assessment of insulin resistance (HOMA-IR) was calculated from fasting plasma glucose and plasma insulin using HOMA2 calculator version 2.2.3 (Diabetes Trials Unit, University of Oxford). Framingham risk score was calculated as described earlier [28]

### Analyses of oxidative stress parameters

The antioxidant capacity of serum was measured via the ferric reducing ability potential (FRAP) assay as described by Benzie and Strain [29] in triplicates using trolox as standard. Absorbance was measured with BMG FLUOstar OPTIMA Microplate Reader (BMG LABTECH GmbH) at 593 nm and results are expressed as trolox equivalents in μmol/L.

MDA levels were determined in duplicates in plasma as described earlier [30]. After heating (60min, 100°C) plasma samples were neutralized with methanol/NaOH, centrifuged (3min, 3000rpm) and MDA was measured with high-performance liquid chromatography (HPLC) (excitation: *λ* 532nm, emission: *λ* 563nm, LaChrom Merck Hitachi Chromatography System, Vienna, Austria; HPLC column 125×4 mm, 5 μm; Merck, Vienna, Austria).

GSSG and GSH were analyzed with use of N-Ethylmaleimide and O-phthalaldehyde according to an adopted method of Hissin and Hilf [31] as described previously [25]. All samples were analyzed fluorometrically in triplicates with external standards of GSSG and GSH using BMG FLUOstar OPTIMA Microplate Reader (BMG LABTECH GmbH).

Reduced thiols as a consequence of oxidative reactions can be quantified by spectrophotometer using 5.5’dithiobis 2-nitrobenzoic acid [32], as described by Hawkins et al. [33]. Serum samples were analyzed in triplicates using GSH as external standard (BMG FLUOstar OPTIMA Microplate Reader, BMG LABTECH GmbH).

Quantification of oxLDL was performed in duplicates with a customary ELISA kit (ox-LDL/MDA Adduct, ELISA, Immundiagnostik) according to manufactures’ instructions.

Total F2-Isoprostanes were extracted from plasma and analyzed in duplicates using gas chromatography tandem mass spectrometry as described previously [34].

### Analyses of antioxidant enzyme activities

The activities of superoxide dismutase (SOD), catalase (CAT) and glutathione peroxidase (GSH-Px) in erythrocytes were analyzed as reported previously [35–37]. For SOD, the inhibition of auto-oxidation of pyrogallol which occurs in the presence of superoxide anion was measured [35]. The activity of CAT was analyzed photometrically by assessing the rate of hydrogen peroxide degradation [36]. The GSH-Px activity was measured using an indirect coupled assay and defined as proportion to the oxidation of 1nmol of nicotinamide adenine dinucleotide phosphate per minute [37]. Antioxidant enzyme activities are presented in units.

### Detection of oxidative DNA damage

Oxidative damage to DNA was measured with single cell gel electrophoresis (comet assay). The comet assay for PBMC was performed adjusted to the method described by Azqueta et al. [38] in a 12-minigel format, as described by the same author [39]. The comet assay for whole blood was performed according to Al-Salmani et al. [40] with slight modifications. Stored whole blood was thawed quickly, 10 μl whole blood was mixed with 200 μl 1%-agarose solution and 5 μl were pipetted on the respective spot of the 12-minigel slide. The same procedure was applied to thawed and washed PBMC samples, whereby 15 μl cell solution with a concentration of 5x10^5^ cells/mL (in phosphate buffered saline solution) was mixed with 70 μl 1%-agarose solution and 5 μl were pipetted on the respective spot of the 12-minigel slide. Each sample was analyzed in duplicates, resulting in 6 different samples per 12-minigel slide. For each 6-sample-group, 3 slides were necessary for the following treatments: lysis, buffer and formamidopyrimidine—DNA glycosylase (FPG), for PBMC and whole blood respectively. All following steps were done equally for whole blood and PBMC slides.

Slides were placed in lysis solution (2.5 M NaCl, 0.1 M EDTA, 10 mM Tris base with 1% Triton X-100, pH 10) for one hour. After lysis, FPG and buffer slides were washed three times with cold enzyme buffer (40 mM HEPES, 0.1 M KCL, 0.5 mM EDTA, 0.2 mg/mL BSA, pH 8) before being clamped into slide units (12-Gel Comet Assay Unit^™^, Severn Biotech Limited). The units were placed on ice and gels were respectively treated with either 30 μL enzyme buffer or 30 μL FPG solution (1:3000 dilution; FPG, New England Biolabs GmbH). The units were hermetically closed, placed in a pre-heated moist box and incubated for 30 min at 37°C. Thereafter, all slides were put in cold electrophoresis solution (0.3 M NaOH, 1 mM EDTA, pH>13) for 20 min unwinding phase, followed by 30 min of electrophoresis (25 V, 300 mA at 4°C). All slides were washed with phosphate buffered saline solution and distilled water. For drying of the gels, slides were first placed in 70% ethanol and second in ethanol absolute for 15 min respectively. Gels were stained with GelRed (PAGE GelRed Nucleic Acid Gel Stain, Biotium). DNA damage was expressed in % Tail DNA which was quantified using a fluorescence microscope (Nikon) and the imaging software Comet Assay IV (Perceptive Instruments Ltd). For each sample 100 cells were scored (50 per duplicate) and means were calculated. FPG-sensitive sites were expressed as net % Tail DNA.

### Detection of DNA BER capacity with the comet repair assay

The DNA BER capacity was observed with the comet-based in vitro repair assay described by Azqueta et al. [41]. Briefly, a substrate was prepared using PBMC from a healthy donor, which was treated with photosensitizer RO 19-8022 and 5 min light-induction to induce 8-oxoguanine in its DNA. A second batch of substrate PBMC was treated the same way excluding RO 19-8022 and therefore served as control. These substrates were the initial substances for the 12-minigel based comet assay approach and embedded in 1%-agarose. After one-hour lysis treatment the gel-embeded cells of the substrate were incubated with PBMC extract for 30 min in a moist box at 37°C. For extract preparation the frozen cell pellet of 5x10^6^ PBMC of each study participant was assimilated in buffer solution as described earlier [41]. After extract incubation, all following comet steps were done as described above for the comet assay, including unwinding phase, electrophoresis, ethanol desiccation, staining and quantification of DNA Tails. Repair-related DNA incisions were calculated according to Azqueta et al. [41] and expressed as % Tail DNA.

### Gene expression analyses

For gene expression analyses, a subset of 46 participants was created. Twenty-three T2DM females with an HbA1c≤7.5% were matched according to age, medication and smoking history to twenty-three T2DM females with HbA1c>7.5%.

RNA was extracted from PBMC using a commercially available extraction Kit (ReliaPrep^™^ RNA Cell Miniprep System, Promega GmbH), quantified (Nanodrop 2000, Thermo Fisher Scientific) and stored at -80°C. Transcription into cDNA was performed with a customary kit according to manufactures’ instructions (QuantiTect^®^ Reverse Transcription Kit, Qiagen). Quality of RNA and cDNA was checked on a random basis using gel-electrophoresis. For gene expression analyses, quantitative real-time polymerase chain reaction (qPCR) was performed using SYBR green-based gene expression assay (SYBR^®^ Select Master Mix, Applied Biosystems^™^, Thermo Fisher Scientific) on a 384-well QuantStudio^™^ 6 Flex Real-Time PCR System (Applied Biosystems^™^, Thermo Fisher Scientific). Primers of nine candidate genes, connected to BER of DNA, and four housekeeping genes (glyceraldehydes-3-phosphate dehydrogenase, *GAPDH*; hypoxanthine phosphoribosyltransferase 1, *HPRT1*; beta-2-microglobulin, *B2M*; Actin beta, *ACTB*) were designed via NCBI Primer Blast, ordered at Sigma-Aldrich and pretested in terms of sequence quality (Table 1). All samples were analyzed on one plate for each gene of interest to minimize inter-plate variations. All results were evaluated as one experiment, including a common threshold using data analysis software for qPCR (Thermo Fisher Cloud, Thermo Fisher Scientific). M-score analyses of housekeeping genes (*GAPDH*, *ACTB*, *HPRT*, *B2M*) were done using geNorm calculation within the Thermo Fisher Cloud qPCR analysis software resulting in *GAPDH* exclusion. The geometric mean of the cycle of threshold value of *ACTB*, *HPRT*, *B2M* was used as reference gene for calculating the relative quantification with the Livak Method [42]. To obtain fold changes of gene expression patients with HbA1c≤7.5% served as control to their respectively matched patients with HbA1c>7.5%.

**Table 1:** Primer sequences for gene expression analyses.

Gene symbol	Gene ID	Primer sequence	bp	Product bp
APEX1	328	Forward: CGGACAAGGAAGGGTACAGT	F 20	83
		Reverse: CTCCTCATCGCCTATGCCGTA	R 21	
LIG3	3980	Forward: AGAGCGAGTCCAGGTGCATA	F 20	88
		Reverse: GTGGGCCACCTTGTGAGGAA	R 20	
MUTYH	4595	Forward: TCCACCGCCATGAAAAAGGT	F 20	77
		Reverse: TGGGACCTTTTGGAACCCATA	R 21	
NEIL1	79661	Forward: GACAGAGTGGAGGACGCTTT	F 20	91
		Reverse: GCTGGGTTGCAGTCCTCTTA	R 20	
NTHL1	4913	Forward: CAGACAGATGATGCCACGCT	F 20	70
		Reverse: TGTATTTCACCTTGCTCCTCCA	R 22	
OGG1	4968	Forward: CCGAGCCATCCTGGAAGAAC	F 20	129
		Reverse: CAGATGCAGTCAGCCACCTTG	R 21	
PARP1	142	Forward: CCACACACAATGCGTATGACT	F 21	113
		Reverse: CCACAGCAATCTTCGGTTATGA	R 22	
POLB	5423	Forward: AAAAGTGGATTCTGAATACATTGCTA	F 26	123
		Reverse: GGCTGTTTGGTTGATTCTGAAG	R 22	
XRCC1	7515	Forward: AAGAAGACCCCCAGCAAACC	F 20	77
		Reverse: CGAGTTGGAGCTGGCAATTT	R 20	
ACTB	60	Forward: TGGCACCCAGCACAATGAA	F 19	183
		Reverse: AGTCATAGTCCGCCTAGAAGCA	R 22	
B2M	567	Forward: CACCCCCACTGAAAAAGATGAG	F 22	106
		Reverse: CCTCCATGATGCTGCTTACATG	R 22	
HPRT1	3251	Forward: TGCTTTCCTTGGTCAGGCAG	F 20	110
		Reverse: TTCAAATCCAACAAAGTCTGGC	R 22	

### Statistical analyses

All statistical analyses were performed with SPSS Statistics software version 22 (International Business Machines Corporation, IBM). Normal distribution was analyzed with Kolmogorov-Smirnov test. Differences between the groups (HbA1c≤7.5% vs. HbA1c>7.5%) were tested with t-test for independent variables or Mann-Whitney-U test for nonparametric variables. Pairwise comparisons between multiple groups were analyzed with one-way analysis of variance (Anova) with Bonferroni adjustment and adjustment of covariates (ANCOVA) if necessary. If normal distribution was not given, the Kruskal-Wallis test was used. Correlations were analyzed with Pearson’s correlation coefficient or Spearman correlation for nonparametric variables. Fold-changes of gene expression were tested with one-sample t-test against “1” or Wilcoxon test against “1” if normal distribution was not present. Significance was assumed at p<0.05.

## Results

### Characteristics of study population

In total 146 T2DM female patients with a mean age of 67.5 (min: 40.0/ max: 86.0) years and a mean BMI of 35.0 (18.9/ 61.2) kg/m^2^ completed the study. On average, the patients have been diagnosed with T2DM for 14.4 (0.1/54.0) years and had a mean HbA1c level of 7.8 (5.9/16.3) %. Out of the 146 subjects, 60 patients received insulin therapy, while 86 patients got other oral antidiabetics or injectables including Metformin, Sulphonylurea, Glinides, Glitazones, Alpha-glucosidase inhibitors, DPP-4 inhibitors, and SGLT2 inhibitors. 60.9% of the participants took medications for altering lipid metabolism and 81.5% antihypertensives. Diabetes complications affecting eyes, kidneys, gum, neural, cardiovascular system as well as chronic inflammation were reported by 44.5% of all patients.

### Differences between HbA1c groups in anthropometrics and clinical biochemistry

Patients with HbA1c≤7.5% (n = 74) had a mean HbA1c of 6.86±0.5% (mean±standard deviation). A significant difference in HbA1c level was found in comparison to the 72 patients representing the HbA1c>7.5% group with 8.69±1.3%. Neither age distribution between the groups with 68.7±9.8 years in the low vs 66.2±10.0 years in the high HbA1c group, nor the DD with 13.6±8.8 years in the low vs 15.2±7.1 years in the high HbA1c group differed significantly (Table 2). Anthropometric parameters such as BMI and systolic blood pressure were significantly higher in patients with higher HbA1c. In contrast, waist-to-hip ratio and diastolic blood pressure did not differ between the groups. Highly significant disparities between the HbA1c groups were found in lipid parameters, resulting in higher total cholesterol (p<0.05), LDL-cholesterol, triglyceride and lower HDL-cholesterol values (p≤0.01), and a higher LDL/HDL-cholesterol ratio (p<0.001) in the high HbA1c group (Table 2). Additionally, significant correlation between HbA1c and total cholesterol (r = 0.328, p≤0.001), LDL-cholesterol (r = 0.336, p≤0.001), triglycerides (r = 0.251, p≤0.001) and HDL-cholesterol (r = -217, p≤0.01) were found. Furthermore, the Framingham risk score was higher in patients with HbA1c>7.5% compared to the low HbA1c group (p<0.01).

**Table 2:** Differences between HbA1c groups in age, anthropometric parameters and clinical biochemistry.

	HbA1c≤7.5%	HbA1c>7.5%	
	Mean ± SD	Mean ± SD	*p-value*^1^
n	74	72	
Metformin therapy [%]	81.1	73.6	
Insulin therapy [%]	28.4	54.2	
Other antidiabetic medication* [%]	71.6	73.6	
Diabetes complication^ǂ^ [%]	46.0	43.0	
Age [years]	68.66 ± 10	66.22 ± 10	.*138*
BMI [kg/m^2^]	33.69 ± 7.5	36.41 ± 7.5	**.*030***
WHR	0.88 ± 0.0	0.89 ± 0.1	.*308*
HbA1c [%]	6.86 ± 0.5	8.69 ± 1.3	**.*000***
Fasting plasma glucose [mmol/L]	7.93 ± 1.7	10.08 ± 2.0	**.*000***
Diabetes duration [years]	13.59 ± 8.8	15.24 ± 7.1	.*219*
Fasting insulin [pmol/L]	114.3 ± 94	141.3 ± 155	.*204*
C-peptide [nmol/L]	1.03 ± 0.5	1.02 ± 0.7	.*907*
HOMA-IR	2.97 ± 2.6	2.19 ± 1.5	.*067*
Blood pressure systolic [mmHg]	137.7 ± 20	145.6 ± 19	**.*014***
Blood pressure diastolic [mmHg]	81.74 ± 11	82.74 ± 10	.*578*
Total cholesterol [mmol/L]	4.15 ± 0.6	4.47 ± 1.0	**.*019***
HDL-cholesterol [mmol/L]	1.45 ± 0.4	1.28 ± 0.3	**.*005***
LDL-cholesterol [mmol/L]	2.03 ± 0.6	2.34 ± 0.8	**.*005***
LDL/HDL-cholesterol	1.49 ± 0.5	1.90 ± 0.7	**.*000***
Triglycerides [mmol/L]	1.48 ± 0.8	2.03 ± 1.8	**.*011***
Framingham risk score [%]	11.63 ± 5.9	14.77 ± 7.3	**.*008***

^1^ differences between groups were analyzed with t-test for independent variables or Mann Whitney U test for nonparametric variables;

* including: Sulphonylurea, Glinides, Glitazones, Alpha-glucosidase inhibitors, DPP-4 inhibitors, SGLT2 inhibitors;

^ǂ^ Diabetes complication according to medical history including: eye, kidney, gum, neural, chronic inflammation and cardiovascular system

HbA1c, glycated hemoglobin; BMI, body mass index; WHR, waist-to-hip-ratio; SD, standard deviation

### Differences between HbA1c groups in oxidative stress parameters, antioxidant enzyme activities, DNA damage and BER capacity

In contrast to anthropometric data and lipid metabolism, no differences between the HbA1c groups were found in parameters concerning oxidative stress, antioxidant enzyme activities, DNA damage or DNA BER capacity (Table 3). Oxidative stress marker such as FRAP, MDA, oxLDL, reduced thiols, GSSG/GSH or F2-Isoprostane were not different between T2DM patients with high or low HbA1c. Similar results were found for the antioxidant enzyme activities of SOD, CAT and GSH-Px. In addition, no distinctions were found in comet assay analyses of PBMC and whole blood including strand breaks and FPG-sensitive sites. The DNA BER capacity in T2DM patients with HbA1c≤7.5% was 12.34±3.5% Tail DNA which was not different from the hyperglycemic T2DM patients with a DNA BER capacity of 11.84±3.8% Tail DNA (Table 3). The results remained unchanged when BMI was considered as covariate in the statistical model. In addition, no correlations between oxidative stress parameters, antioxidant enzyme activities, DNA damage and BER capacity with BMI were observed (data not shown).

**Table 3:** Differences between HbA1c groups in DNA damage, BER capacity, oxidative stress parameters and antioxidant enzyme activities.

	HbA1c≤7.5% (n = 74)	HbA1c>7.5% (n = 72)	
	*Mean ± SD*	*Mean ± SD*	*p-value*^1^
**DNA damage (PBMC)**			
Strand breaks [% Tail DNA]	6.36 ± 3.0	6.36 ± 3.8	.*998*
FPG-sensitive sites [% Tail DNA]	4.50 ± 3.0	4.61 ± 2.7	.*820*
**DNA damage (whole blood)**			
Strand breaks [% Tail DNA]	8.09 ± 5.3	8.77 ± 5.3	.*437*
FPG-sensitive sites [% Tail DNA]	5.82 ± 5.0	5.59 ± 4.0	.*759*
**DNA repair**			
repair capacity [% Tail DNA]	12.34 ± 3.5	11.84 ± 3.8	.*417*
**Oxidative stress marker**			
FRAP [μmol/L]	359.5 ± 127	391.9 ± 228	.*290*
Malondialdehyde [μmol/L]	1.02 ± 0.4	1.06 ± 0.4	.*594*
reduced thiols [μmol/L]	532.4 ± 92	512.7 ± 87	.*185*
GSSG [μmol/L]	9.50 ± 1.5	9.60 ± 1.6	.*721*
GSH [μmol/L]	13.89 ± 2.2	13.77 ± 2.2	.*746*
GSSG / GSH	0.70 ± 0.2	0.71 ± 0.2	.*744*
oxLDL [ng/ml]	151.8 ± 149	156.1 ± 202	.*844*
F2-Isoprostane [pg/ml]	209.0 ± 95	211.7 ± 90	.*615*
**Enzymes**			
SOD [IU/g Hb]	1674 ± 295	1667 ± 255	.*701*
CAT [IU/g Hb]	16.87 ± 4.6	17.48 ± 3.8	.*382*
GSH-Px [IU/g Hb]	34.14 ± 6.5	35.31 ± 7.6	.*318*

^1^ differences between groups were analyzed with t-test for independent variables or Mann-Whitney-U test for nonparametric variables

HbA1c, glycated hemoglobin; FRAP, ferric reducing ability potential; GSSG, oxidized glutathione; GSH, reduced glutathione; oxLDL, oxidized low-density lipoprotein; SOD, superoxide dismutase; CAT, catalase; GSH-Px, glutathione peroxidase

### Differences between DD in oxidative stress parameters, antioxidant enzyme activities, DNA damage and BER capacity

The distribution of DD within the patients differed substantially from newly diagnosed to a maximum of 54.0 years. Within the DD tertiles (DD I = 6.94±3.1 y, n = 49, DD II = 13.35±1.1 y, n = 48, DD III = 22.90±7.3 y, n = 49), there was a significant increase in age with rising DD. In addition, differences in HbA1c between DD I and DD III (p = 0.008) and a trend between DD II and DD III (p = 0.062) were present. However, no differences were found in most oxidative stress parameters, antioxidant enzyme activities, DNA damage and BER capacity between the three DD groups (Table 4). Only F2-Isoprostane was significantly different between the DD groups with a slight increase between DD I and DD II (p = 0.054). However, it was not seen any longer after age-adjustment. Other results remained unchanged when age was taken as covariate in the statistical model.

**Table 4:** Differences between DD groups in DNA damage, BER capacity, oxidative stress parameters and antioxidant enzyme activities.

	DD I	DD II	DD III	
	(n = 49)	(n = 48)	(n = 49)	
	*Mean ± SD*	*Mean ± SD*	*Mean ± SD*	*p-value*^1^
Diabetes duration [years]	6.94 ± 3.1^a^	13.35 ± 1.1^b^	22.90 ± 7.3^c^	.*000*
Age [years]	63.02 ± 10.2^a^	67.69 ± 9.5^b^	71.67 ± 8.2^b^^c^	.*000*
HbA1c [%]	7.65 ± 1.7^a^	7.54 ± 0.9^a^^b^	8.11 ± 1.1^b^	.*007*
**DNA damage (PBMC)**				
Strand breaks [% Tail DNA]	6.65 ± 2.9	5.73 ± 2.6	6.68 ± 4.4	.*226*
FPG-sensitive sites [% Tail DNA]	4.03 ± 2.6	4.55 ± 2.6	5.09 ± 3.3	.*189*
**DNA damage (whole blood)**				
Strand breaks [% Tail DNA]	7.70 ± 5.6	8.72 ± 5.1	8.87 ± 5.1	.*262*
FPG-sensitive sites [% Tail DNA]	6.28 ± 5.3	6.34 ± 4.7	4.51 ± 3.1	.*167*
**DNA repair**				
repair capacity [% Tail DNA]	12.25 ± 3.3	11.65 ± 3.8	12.37 ± 3.9	.*514*
**Oxidative stress marker**				
FRAP [μmol/L]	358.0 ± 129	370.3 ± 128	398.0 ± 262	.*844*
Malondialdehyde [μmol/L]	1.05 ± 0.4	1.06 ± 0.5	0.99 ± 0.4	.*794*
reduced thiols [μmol/L]	520.5 ± 92	522.4 ± 88	525.0 ± 90	.*970*
GSSG [μmol/L]	9.46 ± 1.4	9.51 ± 1.6	9.67 ± 1.6	.*789*
GSH [μmol/L]	14.20 ± 2.0	13.82 ± 2.5	13.47 ± 2.0	.*186*
oxLDL [ng/ml]	152.8 ± 152	161.2 ± 226	147.2 ± 138	.*905*
F2-Isoprostane [pg/ml]	181.4 ± 77	226.0 ± 95	223.9 ± 99	.*031*
**Enzymes**				
SOD [IU/g Hb]	1692 ± 265	1662 ± 236	1658 ± 322	.*788*
CAT [IU/g Hb]	17.47 ± 4.7	17.46 ± 4.3	16.59 ± 3.5	.*493*
GSH-Px [IU/g Hb]	36.01 ± 7.2	34.19 ± 7.3	33.94 ± 6.7	.*289*

^1^ differences between groups were analyzed with one-way Anova with pairwise comparisons or Kruskal-Wallis test with pairwise comparisons for nonparametric variables;

^a,b,c^ indicate differences between groups

HbA1c, glycated hemoglobin; FRAP, ferric reducing ability potential; GSSG, oxidized glutathione; GSH, reduced glutathione; oxLDL, oxidized low-density lipoprotein; SOD, superoxide dismutase; CAT, catalase; GSH-Px, glutathione peroxidase

### Gene expression of BER enzymes

**Fig 1:**
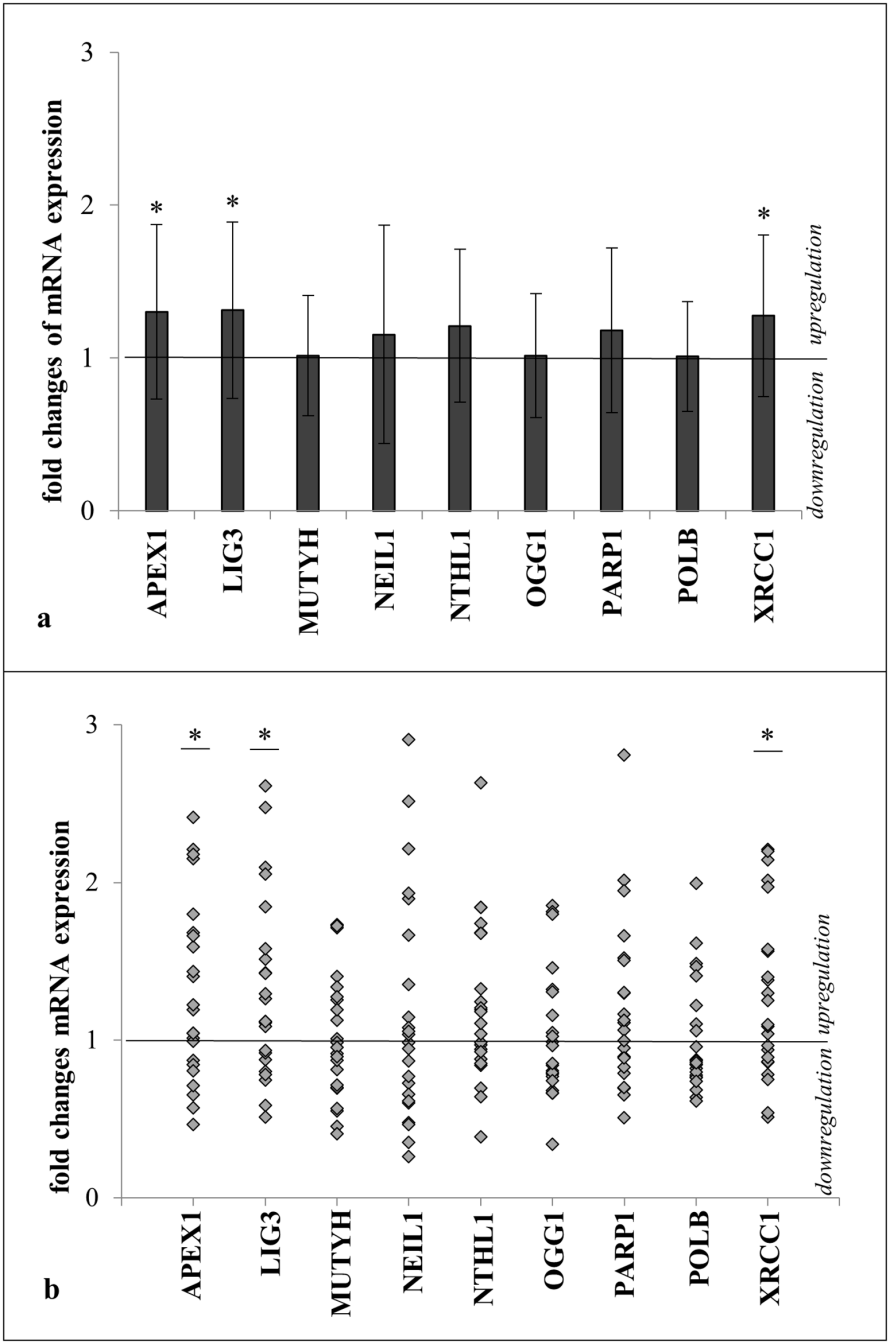
Fold changes of mRNA expression of DNA BER enzymes. T2DM patients with HbA1c>7.5% (n = 23) in relation to matched T2DM patients with HbA1c<7.5% (n = 23). For each pair, results were normalized to the HbA1c<7.5% expression. Matching was according to age, medication and smoking history. Significance was assumed at p<0.05 and tested with one-sample t-test against “1” or Wilcoxon test against “1” if normal distribution was not assumed. (a) Fold-changes presented as bar plots showing mean and standard deviation. (b) Distribution of fold changes. Each point represents a matching pair (n = 23).

To gain a further insight into DNA repair capacity, mRNA expression of nine enzymes involved in BER was measured in a subset of 46 matched (according to age, medication and smoking history) T2DM patients with either high or low HbA1c (cut off 7.5%) (Table 1). A significant up-regulation was found in *APEX1* (fold change: 0.30, p = 0.018), *LIG3* (fold-change: 0.31, p = 0.016) and *XRCC1* (fold-change: 0.28, p = 0.02) in patients with higher HbA1c compared to their matched partners with lower HbA1c (Fig 1a). In addition, a strong correlation between *XRCC1* and *LIG3* (r = 0.668, p = 0.000) and *APEX1* (r = 0.709, p = 0.000) was observed. Other genes involved in BER including *MUTYH*, *NEIL1*, *NTHL1*, *OGG1*, *POLB* and poly(ADP-ribose) polymerase 1 (*PARP1*) did not result in fold-change differences in high vs. low HbA1c groups (Fig 1a). However, regarding the huge distribution of fold-changes within the 23 matched pairs, some patients showed a high up-regulation of BER genes (Fig 1b).

## Discussion

The present study with 146 female T2DM patients, with HbA1c levels either ≤7.5% or >7.5% and three different DD groups did not show major differences in oxidative stress parameters, antioxidant enzyme activities, DNA damage or DNA repair. Therefore our results do not confirm the assumption that oxidative stress and its resulting damage to DNA are increasing with T2DM progression. Most previous studies compared healthy individuals to T2DM patients and could detect induced oxidative stress in T2DM, represented through decreased FRAP-, GSH- and reduced thiols levels, whereas MDA, oxLDL, GSSG and F2-Isoprostanes were increased [20–24, 43, 44]. Additionally, strong linear association to HbA1c was reported for MDA, GSH and oxLDL [20, 45]. However, the correlation analyses (data not shown) in our study population did not show any significant associations between HbA1c and oxidative markers. Regarding antioxidant enzyme activities of SOD, CAT, GSH-Px, we could not detect any differences between the HbA1c groups. SOD and CAT activity was previously shown to be increased in T2DM compared to healthy controls [20, 43, 46, 47], while GSH-Px was decreased [20, 47]. However, evidence is not sound as some studies could not detect any differences between T2DM and healthy controls in these enzymes [46, 47].

Regarding the progression of the disease over time, no differences in oxidative stress markers could be found between the DD tertiles. Nakhjavani et al. [48] showed that newly diagnosed T2DM patients without any treatment had lower oxLDL levels compared to T2DM patients with a DD>5 y. In the present study newly diagnosed patients are rare and all patients were under medical treatment. It can be assumed that despite the different glycemic states and large distribution in DD within the study population, the good medical treatment and regular biannual medical observations protect against major redox deregulation within the patients.

Increased oxidative stress and its resulting reactive oxygen species are some of the leading causes for accumulation of DNA damage [11]. Because of the lacking connection between hyperglycemia and induced oxidative stress, it is not surprising that DNA damage parameters did not differ between T2DM patients with HbA1c≤7.5% or >7.5%. Additionally, published data about DNA damage in T2DM are also inconsistent and while some studies reported a clear increase in DNA damage in T2DM patient vs. healthy controls [18, 19] other studies could not detect any differences [26, 49]. To our knowledge there are only few other studies who analyzed DNA damage in T2DM with either low or high HbA1c. Xavier et al. [50] detected higher damage to DNA in hyperglycemic (HbA1c>7%) T2DM patients compared to non-hyperglycemic (HbA1c<7%) T2DM individuals using the comet assay in PBMC. Another study found higher FPG-sensitive sites in poorly-controlled T2DM with higher HbA1c compared to well-controlled individuals [51]. Furthermore, a strong direct correlation between DNA damage and HbA1c was found in 427 T2DM patients in the study of Choi et al. [52]. However, these studies did not analyze oxidative stress parameters to connect detected DNA damage to its possible origin. Regarding DD, no differences concerning comet assay results were found in the present study. Similar results are shown in a study on 72 Mexican T2DM individuals, with similar age and DD distribution to the present study, where Ibarra-Costilla et al. [49] were not able to detect differences in DNA damage depending on DD.

A lack of increased DNA damage could also be explained by an improved DNA repair capacity. However, BER capacity measured by the comet repair assay did not result in differences between the HbA1c groups. A closer look into gene expression analysis of nine involved BER genes of 46 matched T2DM patients revealed a statistically significant up-regulation of *APEX1*, *LIG3* and *XRCC1* of T2DM with higher HbA1c. *LIG3* and *XRCC1* build a protein complex during BER [53]. In addition, *XRCC1* interacts with other BER involved proteins, including *APEX1*, *POLB* and *OGG1*, and is therefore involved in almost every step of BER [54]. Due to their common interaction, it was not surprising that *XRCC1* and *LIG3*, as well as *APEX1* showed similar expression behavior and a strong correlation. DNA repair capacity in T2DM patients compared to healthy controls is reported to be lower [16, 18] and gene expression analyses by microarray showed down-regulation of DNA repair genes in T2DM individuals [17]. Interestingly, we found that *APEX1*, *LIG3* and *XRCC1* transcription is induced in T2DM patients with higher HbA1c resulting a 30% up-regulation compared to their matched T2DM with low HbA1c. A very recent study by Xavier et al. [50] also found induced gene expression in T2DM with higher HbA1c compared to T2DM with lower HbA1c in an entire gene set representing DNA repair in their study using microarray analysis. They explained it as a compensatory mechanism to higher DNA damage in their high-HbA1c group [50]. In the present study no difference was found in DNA damage between high- or low-hyperglycemic patients but we still observed an increased mRNA expression in three important BER related genes. However, as the up-regulation was only 30%, even if statistically significant, it might not necessarily lead to a meaningful biological outcome. Further studies focusing on protein levels and functional assays to evaluate the BER capacity of T2DM are needed to completely understand the connection of hyperglycemia and DNA repair and its influences on secondary diabetes complications.

Although our main parameters of interest did not reveal major outcomes, there was a difference in biochemical parameters between patients with higher HbA1c to well-controlled patients with lower HbA1c, especially regarding blood lipid parameters and blood pressure. We found higher total cholesterol, LDL-cholesterol, LDL/HDL-cholesterol, triglycerides, Framingham risk score, systolic blood pressure, BMI and lower HDL-cholesterol in hyperglycemic patients with HbA1c>7.5%. These parameters are all known risk factors for cardiovascular events which still is one of the leading causes of death in T2DM [10]. Given the fact that 60.9% of the patients took medical treatment concerning blood lipid regulation and 81.5% against hypertension, the remaining differences are alarming and could result in an earlier diabetes-related death in the high HbA1c group.

Several points might be considered as a drawback of the study. First, the exclusion of male gender which was due to an intended homogenous study population. Men and women differ in regard to metabolic pathways and insulin sensitivity [55] and have different hormonal statuses which might have led to different outcomes in the parameters tested. Learned from a recent study females are easier to recruit, since they are more reliable and dedicated [26]. Therefore, it was a conscious decision to exclude male gender to create a study population which was as homogenous as possible and mainly differed in HbA1c and DD. Second, the narrow distribution of the HbA1c values could be regarded critically. All of the patients were under medical treatment concerning blood glucose management and regular medical observations, not allowing major discrepancies between the HbA1c groups. Third, the regular medical treatment of the subjects was not only focused on hyperglycemia but in many cases also against hypertension, hyperlipidemia and other metabolic imbalances. Regarding the mechanism of action, not only Metformin but also Statins have been discussed previously to act in an antioxidative manner with DNA-damage-protecting properties [56–58]. Thus, they could be regarded as confounding factors in the assessment of oxidative stress, antioxidant enzyme activities, DNA damage and DNA repair in medically controlled individuals with T2DM. However, it also reflects the positive situation in an industrialized western country such as Austria where the health system offers regular blood glucose controls and individually optimized medical treatment for T2DM patients.
